# Percutaneous Dilatational Tracheostomy and Surgically Created Thracheostomy in ICU Patients

**DOI:** 10.5681/jcvtr.2014.008

**Published:** 2014-03-21

**Authors:** Mohammad Amin Valizade Hasanloei, Alireza Mahoori, Amir Mohammad Bazzazi, Samad EJ Golzari, Tohid Karami

**Affiliations:** ^1^Department of Anesthesiology and Intensive Care Medicine, Urmia University of Medical Sciences, Urmia, Iran; ^2^Department of Neurosurgery, Urmia University of Medical Sciences, Urmia, Iran; ^3^Cardiovascular Research Center, Tabriz University of Medical Sciences, Tabriz, Iran

**Keywords:** Percutaneous Dilatation Tracheostomy (PDT), Surgically Created Tracheostomy (SCT), Complications, Intensive Care Unit

## Abstract

***Introduction:*** Following advances of Intensive Care medicine and widespread administration of mechanical ventilation, tracheostomy has become one of the indispensable surgical procedures. During this research we tried to assess and compare two main strategies for doing tracheostomy: Surgically Created Tracheostomy (SCT) and Percutaneous Dilatational Tracheostomy (PDT).

***Methods:*** In a randomized clinical trial, 60 cases of patients who were admitted in Intensive Care Unit (ICU) and needed tracheostomy during their stay were enrolled. Patients were randomly divided into two groups. SCT technique was considered for the first group and PDT for the second one. Demographic characteristics, associated and underlying diseases, type and duration of procedure, duration of receiving mechanical ventilation and ICU stay, expenses and complications of tracheostomy including bleeding, subcutaneous emphysema, pneumothorax, stomal infection and airway loss were all recorded during study and compared between both groups.

***Results:*** There were significant differences between two groups of patients in terms of duration of receiving mechanical ventilation (P=0.04), duration of tracheostomy procedure (P=0.001) and procedure expenses (P=0.04). There was no significant difference between two groups in terms of age and gender of patients, duration of ICU stay and complications of tracheostomy including copious bleeding, stomal infection, subcutaneous emphysema and airway.

***Conclusion:*** According to the results of our study and similar researches, it can be concluded that PDT can be considered as the preferred procedure in cautiously selected patients during their ICU stay.

## Introduction

Tracheostomy has been performed by physicians for centuries in order to save lives and secure airway.^1^Although tracheostomy can be associated with some complications, especially in long term use^2^, it is essential for physicians to know the procedure.^3^ Many patients in ICU need airway management and ventilatory support. Special cases that need ventilatory support through endotracheal tube are:

A. Initiation of mechanical ventilation
B. Airway support
C. Insufficient oxygenation while using less invasive procedures
D. To avoid aspiration and to provide suction of pulmonary secretion
E. Hyperventilation to reduce intracranial pressure^4^

Tracheostomy is indicated in patients who need long intubation period, frequent pulmonary suctions, and those who have airway disorders secondary to neurological defects.^5^

Tracheostomy is opening a stoma towards trachea through the neck by surgical methods that put the tracheal mucosa along the skin. Nowadays, following advances of intensive care medicine and widespread administration of mechanical ventilation, tracheostomy has become one of the indispensable surgical procedures.^6^

Although no distinct guidelines have been defined, tracheostomy is considered in patients predicted to be under mechanical ventilation for more than 3 weeks. Tracheostomy does not obviously reduce laryngeal injury or tracheal stenosis; however, it leads to patient’s comfort and cooperation in rehabilitation activities.^7^

Due to proper humidifying and progressive nursing care and suction, children can tolerate intubating for more than 2 weeks prior to tracheostomy. But tracheostomy is indicated in children who need longer support by mechanical ventilation or intra-tracheal suction or surgical bypass of an upper airway obstruction.^8^ This is a matter of controversy in adults, but after 5-7 days of intubating, the patient is assessed and a decision is made.^9^

The aim of tracheostomy is to minimize chronic airway disorders and tracheal trauma. No distinct time is defined. Tracheostomy is preferred when airway support is predicted to be more than 21 days, but when it is not known exactly, daily visits are necessary to make a decision. Absolute indication of tracheostomy is difficult airway in patients who need a long period of mechanical ventilation. This procedure is performed to facilitate the weaning from mechanical ventilator, reduce infectious complications, promote oral and pulmonary hygiene and patient’s comfort and provide a reliable airway in selected patients and oral feeding and speech.

Traditionally, tracheostomies were performed in operating rooms under surgical standards. In 1985, ciaglia et al, described “percutaneous dilatational tracheostomy”, under a modified seldinger technic using bronchoscopy. This method, widely accepted later, became a procedure of choice in many centers. PDT is simple to learn, and systematically trained persons, perform it confidentially and effective. For satisfactory results, patient selection is important. They should be under minimal ventilatory support (PEEP<7.5 cmH_2_O, FiO2<50%) with a normal coagulation profile (INR<1.3, plt>100000 cell/U) and suitable neck anatomy and external landmarks in neck extension (cricoid cartilage and sternal notch. PDT is contra-indicated in difficult airway maxillofacial trauma–glottis edema–difficult view of vocal cords) or any situations of difficult trans-laryngeal re-intubating. PDT is an elective procedure and should not be performed in emergent airway attempts. PDT has several potential benefits, and many significant complications with high mortalities, such as insertion around the trachea, laceration and tearing of the trachea and esophagus, pneumothorax, and airway loss. Studies suggest that PDT has post-operative bleeding and infection around the stoma, because of the small space between the tracheostomy and tissue around the trachea, acting as a barrier against bleeding and infection.^10^

It has been demonstrated that PDT is a faster procedure with less post-operative complications than open surgical method.^11,12^

PDT technics are as follows:

1. Guide wire dilating forcepse (GWDF)
2. ULTRA–Perce single–stage dilator A
3. Ciaglia Blue Rhino (CBR)

An equal reliability between the two methods and no meaningful difference in major peri-operative complications have been reported.^13,14^ In a recently performed meta-analysis, different PDT methods were compared and it was noted that “single stage dilator A” method was associated with less complications.^15^

With the intention of not transferring the patients outside the ICU for para-clinic and invasive interventions, PDT is performed in the ICUs as an accepted and new method. Hence, we decided to compare the outcomes of PDT with SCT in a clinical trial study.

## Materials and methods

In a randomized clinical trial and after approval of the ethics committee of the university, 60 hospitalized patients in the ICU of Imam Khomeini hospital, Urmia, Iran who needed tracheostomy were enrolled to our clinical trial study in two equal groups of PDT and SCT. Informed written consents were obtained from the patients or their guardians. Later, they were enrolled using random numbers table, and outcomes and complications of each group were compared. As previously mentioned, PDT was performed in patients who were under minimal ventilatory support, had normal coagulation, and proper neck landmarks in extended neck. Patient with difficult airway, maxillo-facial trauma, glottic edema, difficult view of vocal cords and any possibilities of difficulties in trans-laryngeal intubating or emergent tracheostomy necessity were excluded from the study.

After clinical and laboratory evaluation, the eligible ICU patients of PDT group were positioned on their beds under close monitoring of systolic and diastolic blood pressure, heart rate, O_2_ sat, and ECG. Then, under mechanical ventilation, general anesthesia was inducted, laryngoscopy was performed and endotracheal tube was moved to place in the beginning of the larynx below the vocal cords. After fixation of the tube, under sterile condition, anatomic markers of the trachea were detected and tracheostomy was performed by Portex set and Ciaglia Blue Rhino (CBR) method. Then, extubation was performed and mechanical ventilation continued through tracheostomy. For SCT group, they were transferred from ICU to the operating room and tracheostomy was performed surgically under the same monitoring and general anesthesia. After the extubation of the endotracheal tube, mechanical ventilation was continued through tracheostomy. Patients were returned to the ICU after recovery. Obtained data from patients’ medical records were collected from the questionnaires and analyzed by SPSS version 18; Chi-square and T-test were used to evaluate the variables.

## Results

Demographic characteristics of the patients have been presented in Table 1.

**Table 1:** Demographic characteristics of the patients

**Variable**	**PDT**	**SCT**	***P***
Age (years)	64 ±18.64	63.2±21.3	0.84
ICU stay length (days)	51.73±24.37	66.8±31.45	0.08
Mechanical ventilation length (days)	45.87±23.69	58.47±32.72	0.04
Tracheostomy procedure duration (minutes)	4.63±1.67	33.53±8.62	0.001
Cost (Rials)	4000000± 200000	4700000± 35000	0.04

Complications of the patients in both groups have been compared in Table 2. Significant differences could be observed between the two groups in duration of mechanical ventilation (*P*=0.04), duration of tracheostomy procedure (P=0.001), and patient’s costs (*P*=0.04) (Table 2).

**Table 2:** Complications of the patients in both groups

**Variable**	**PDT**	**SCT**	***P***
Average bleeding (mL)	7.5±3	23±18	0.84
Stoma infection	0	3 (10%)	0.08
Subcutaneous emphysema	0	3 (10%)	0.04
Airway loss	1 (3.33%)	2 (6.66%)	0.001
Death	0	1(3.33%)	0.04
Pneumothorax and delayed bleeding	0	0	-

## Discussion

Since 1909, when Jakson described standard method of tracheostomy for the first time, this procedure became the gold standard of airway management in critical patients; however, cricothyroidotomy was considered as the alternative method. In 1950, Shelden introduced the technic of dilatational tracheostomy through the skin; but this method was not welcomed by ICU specialists and surgeons, because of its risk of arterial and esophageal injury. In 1985, Ciaglia described the method of percutaneous Dilatational Tracheostomy (PDT), which was widely accepted and increasingly performed bedside by ENT and other surgeons. Many clinical trials, review and meta-analyses have been conducted to compare tracheostomy methods.^11-22^

Our research showed a statistically significant difference between the two methods of PDT and SCT in terms of the duration of tracheostomy procedure, duration of mechanical ventilation, and procedure expenses; but no meaningful difference in average age, duration of ICU stay, significant bleeding, stoma infection, subcutaneous emphysema, airway loss, and mortality.

In 2012, Kumar et al.^13^ , investigated 30 ICU patients who required tracheostomy and compared the two methods of percutaneous dilatational tracheostomy and their early and late complications. The results showed that duration of tracheostomy in guide wire and forceps method was 6.48±11.68 minutes, and in Ultra perce method 13.93±11.54. Later, the authors concluded that both methods had the same reliability bedside the patient. In our study, PDT duration was 4.63±1.67 minutes and PDT had less complications compared to SCT.

In a survey on overweight patients, Byhahn C et al.^14^ suggested that PDT was associated with more perioperative complications. In our study, 3 of the 30 PDT patients were overweight and had not suitable landmarks for PDT and 1 airway loss and significant bleeding prolonged the time of operation happened. Our study suggests that proper selection of patients with suitable specifications is the best thing to do for reducing the complications.

In an organized meta-analysis on PDT and SCT by Delaney et al.^16^, PDT was reported to be associated with less wound infection, bleeding, and mortality compare to SCT. We had no significant difference in these regards, maybe because of our small sample size (30 cases in each group) versus their 1212 cases in 17 clinical trial studies.

Higgins et al.^17^ in a meta-analysis on about 1000 patients, concluded that PDT is cost-benefit, available bedside, and does not require an operating room. It lasted 4.6 minutes and its expense was 456$. Our study confirms theirs, but our cost difference is not as theirs.

Turkmen et al.^18^ investigated the complications of PDT and SCT by a control MRI one month after closure of the tracheostomy in 30 critical patients. They concluded that PDT seems safer and more effective than SCT. Although we did not consider tracheal stenosis and did not use MRI, the findings of our study are in line with theirs.

Kumar et al.^19^ studied the role of CXR after PDT and concluded that CXR had no changes after non-complicated PDT under the guide of bronchoscopy. Therefore, it seems that the roll of CXR after PDT is limited to those with technical difficulties. In our study, we took CXR in all 30 patients and did not find any complications; however, we did not use bronchoscopy for PDT. So it is the question if CXR is limited to PDT with technical difficulties or not.

Karvandian et al.^20^, compared safety and complications of PDT by Giggs method with SCT in 100 patients. A 5-month follow up period revealed that bleeding, stoma infection, pneumothorax, emphysema, duration of the procedure, and duration of the closure of the tracheostomy was obviously less in PDT compared to SCT group. Therefore, they stated that PDT is the choice method for ICU patients who need tracheostomy. In our study, similarly, PDT was of fewer complications than SCT, but there were no statistically significant differences in mortality.

In a meta-analysis by Satio^21^, 490 PDT patients were compared with 484 OST patients (open surgery tracheostomy). They found that PDT group obviously had less malformed scars, wound and stoma infection, and duration of the procedure. Decannulation complication and airway obstruction were less in OST group, and no difference between the two groups was observed in mortality, bleeding, and airway loss. In another review study, it was represented that duration of the operation and delayed complications of tracheostomy was obviously less in PDT than SCT. Although insertion of the tube was relatively difficult in PDT method, other peri-operation complications did not show any differences, but stoma infection and cosmetic sequelae in SCT group were more than PDT. Our study confirms theirs but we did not consider the airway obstruction.

## Conclusion

PDT is the preferred method of choice in ICU patients who need tracheostomy, provided that the patients have appropriate conditions and are selected cautiously. Future studies with larger sample sizes and longer periods of survey for comparison of delayed, peri-operative, and early complications are recommended.

## Ethical issues

All patients gave written informed consents and the study was approved by our local Ethics Committee.

## Competing interests

The authors of the present work certify that there is no conflict of interest.
